# An Attribute Extraction for Automated Malware Attack Classification and Detection Using Soft Computing Techniques

**DOI:** 10.1155/2022/5061059

**Published:** 2022-04-25

**Authors:** Nabeel Albishry, Rayed AlGhamdi, Abdulmohsen Almalawi, Asif Irshad Khan, Pravin R. Kshirsagar, BaruDebtera

**Affiliations:** ^1^Department of Information Technology, Faculty of Computing and Information Technology, King Abdulaziz University, Jeddah 21589, Saudi Arabia; ^2^Computer Science Department, Faculty of Computing and Information Technology, King Abdulaziz University, Jeddah 21589, Saudi Arabia; ^3^Department of Artificial Intelligence, G.H.Raisoni College of Engineering, Nagpur, India; ^4^Department of Chemical Engineering College of Biological and Chemical Engineering, Addis Ababa Science and Technology University, Addis Ababa, Ethiopia

## Abstract

Malware has grown in popularity as a method of conducting cyber assaults in former decades as a result of numerous new deception methods employed by malware. To preserve networks, information, and intelligence, malware must be detected as soon as feasible. This article compares various attribute extraction techniques with distinct machine learning algorithms for static malware classification and detection. The findings indicated that merging PCA attribute extraction and SVM classifier results in the highest correct rate with the fewest possible attributes, and this paper discusses sophisticated malware, their detection techniques, and how and where to defend systems and data from malware attacks. Overall, 96% the proposed method determines the malware more accurately than the existing methods.

## 1. Introduction

Internet in general is ubiquitous and plays a significant role in our everyday lives. Simultaneously, the Web is constantly vulnerable to security attacks. Malware is one of these dangers; it is defined as malicious software that can exploit security flaws in operating systems and computer services. Without human interaction, malware may propagate rapidly across networks. Malicious authors may create fresh malware variants by using online building packages [1]. Known malicious varieties acquire similar essential capabilities from their progenitors but add extra capability or make nonfunctional changes to their code base. Malware detection is an active research topic due to the fact that malware authors aptly illustrate updated malware versions by using various concealment strategies to avoid current investigative techniques.

It is essential to create proposed detection strategies in order to avoid the creation of new malware kinds. These approaches should be predicated on enhanced exploitation or abnormal detection systems. The issue with anomaly detection techniques is their higher prevalence of alerts. Conversely, traditional exploitation or cryptography diagnosis relies on identifying the fingerprint template associated with each virus [2]. These methods can not detect newly discovered malware strains. Each day, the quantity of these signatures grows, resulting in an increase in the volume of identity collections. This has resulted in an increase in the duration order to match transmissions for signatures. As a result, machine learning has been suggested lately as a method for detecting malware. Machine learning methods are typically focused on discovering and extracting relationships in observed evidence. It has been shown that machine learning can recognize malware samples strains [3]. Artificial intelligence methods are limited by a rising false positive rate, leading to inefficient features extraction strategies, insufficient and repetitive features, and unsuitable classiﬁers generation methods.

Malware has evolved into a potent tool for hackers looking to exploit critical security flaws. As a result, malware manufacturing has risen dramatically during the past 3 decades. Similarly, many attempts have been made to combat malware, and various methods based on signature, behavior, heuristic, application security, and artificial intelligence approaches, among others, have been created [4]. Several studies have suggested various methods for preventing malware using abovementioned approaches. Others, on the other hand, have concentrated their efforts on enhancing the capabilities of harmful software. In this regard, malware research becomes critical since it enables security analysts to comprehend the attacker's viewpoint in order to defend systems against them [3].

The proliferation of malware kinds has resulted in a comeback in terms of reward for infecting regions and technological sophistication. Indeed, malware writers are more orderly and attempt to command the respect of their opponents by remaining silent throughout the infection process, using features such as social engineering or even propagating via the web. The aim of this paper is to give a concise overview of virus identification techniques, which use both traditional and novel approaches, including AI technology [5]. Then, we examine malware evasion methods, including concealment, polymorphism, and GPU-assisted evasion. Finally, based on prior research, we address the use of Machine Learning methods for avoiding anti-malware systems, as well as strategies to combine such techniques.

Nowadays, technology has progressed to the point where everything is moving toward a more digital approach rather than a more mechanical one. Technologies offer both advantages and disadvantages; if it made life simpler, it also attracts cyber assaults, loss of data, and granting access to your personal life to someone who might abuse it. As a result, gadget security is vital in today's cyber environment. Internet use continues to grow at a rapid pace. One disadvantage of extensive Internet usage is that many computer systems are susceptible to assault and infection with malware [6]. Malware is referred to by a variety of terms, including malicious software, harmful programs, and harmful executable. Malware is harmful software that is designed to violate a computer system's security policies on privacy preservation, security, and accessibility. It has the ability to modify and delete your platform's information without your awareness in order to cause damage to the world [4].

## 2. Objective

The main objective of this paper is to use machine learning and create an algorithm that will accomplish the aim of automated malware attack classification and detection using attribute extraction and artificial intelligence methods.

Enhancing the malware epidemic incident response process: if it is determined which malware family it belongs to, what effects it may have on the system, and what precautions should be done to avoid future spread and harm to the framework. Malware analysis: gain a better understanding of how malware acts and the cutting-edge methods used to create it. This will contribute to increasing the accuracy of machine learning models by taking into account newly discovered characteristics. Detecting new indicators of penetration: new indicators of compromise may be utilized in security solutions to assist organizations in defending against malware attacks.

## 3. Review of the Literature

Liu et al. [7] proposed that a machine-learning malware system should be divided into three components. One is the assessment of information and the identification of malware. The other is judgment. The first section discusses the Opcode n-gram and gray-scale pictures, as well as import methods for extracting malware characteristics. Later, they often include capabilities and categories and detected files that seem to be malicious. The database included in this article contains “50,000 malware cases” that were gathered by Anubis, Kingsoft, and ESET NOD32. Finally, the detection section would use the “shared nearest neighbor (SNN) algorithm” to classify the malware samples family, concluding in this method classifying with a rate of 99 percent accuracy and detecting the particular virus with a rate of 87.9 percent.

Souri and Hosseini [8] provide a review of malware detection techniques, categorizing them as signature-based and behavior-based. The study, on the other hand, does not include an assessment of current deep learning approaches or a description of the kinds of attributes utilized in data mining methods for malware identification and tracking.

Ucci et al. [9] classify techniques based on the job they attempt to perform, the attribute categories retrieved from Movable Executable files, and the machine learning algorithms used. While the study describes the feature taxonomy in detail, it does not include emerging trends in research, particularly deep learning and multimodal methods.

Kshirsagar et.al [4] develop the diverse categorization and classification approaches of benchmark functions and real-time monitoring information relevant in all areas and develop hybrid artificially intelligent and optimization methods for the analysis and regression of multiple databases with high precision [10]. In cyberspace, cloud technology, and cloud services, methods were helpful in getting reliable findings using varied assessment criteria [11].

Ye et al. [12] include conventional machine learning techniques for malware detection, including extracting features, feature engineering, and categorization. Nevertheless, critical properties such as a file's entropy or structure entropy, as well as certain dynamic characteristics like as network connections, op code, and API traces, are absent. Additionally, deep learning techniques and multimodal techniques to detecting attacks are not addressed, despite the fact that they have been hot subjects for the past several years.

Fuyong and Tiezhu [13] presented a technique for calculating the gain ratio of each bytes n-gram in the training samples and selecting as features the *K* n-grams with the highest information gain. They then averaged each characteristic of the extracted features extracted from malicious and benign samples individually. Finally, a new piece of software was classified into one of the two groups based on the closeness of the unidentified sample's feature vector to the averaged vector of the 2 categories.

Salehi et al. [14], based on the inputs and return values of API calls, suggested a dynamic technique for detecting malicious behavior in Android APKs. They created an “in-house” solution comprised of a virtual machine, a hooking tool, and a logging system for analyzing and monitoring binary data. Their method is predicated on the premise that API names alone may not adequately convey the meaning of the activities performed by the function. As a result, the feature set modeled dangerous and benign behaviors utilizing API calls, their input parameters, and return values. Following that, the feature set was decreased in two stages. The Fisher score was used in the first step to identify the most discriminating characteristics. The second step used Support Vector Machines with Recursive Feature Elimination to further decrease the set of features. The produced feature set was then sent to the classification techniques as inputs.

Numerous researches on malware detection methods have been conducted; however, there is no approach that fully detects malware, making malware identification difficult. Artificial intelligence methods have been used lately to automated malware categorization and detection in order to combat huge quantities of malware samples. Despite their achievements, they lack the basic fundamental grasp of many critical problems, such as which attribute to utilize and which classifiers work well enough on malware evidence.

## 4. Types of Malware

**Figure 1:**
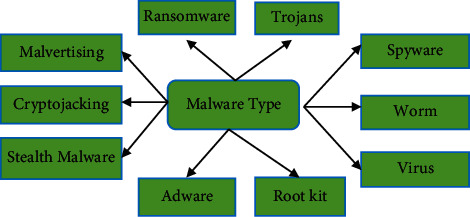
Types of malware.

Figure 1 shows the different types of malware.

### 4.1. Virus

Viruses encrypt their harmful programming and wait for an unwary human or programmed procedure to activate it. As with actual viruses, they may grow rapidly and extensively, wreaking havoc on networks' fundamental functioning, destroying data, and preventing users from using their machines [11]. They often conceal themselves inside an executable program.

### 4.2. Worm

Worms are so named due to the manner in which they infect computers. They thread their course across the system, beginning with one infected device and attaching to subsequent devices in order to propagate disease [15]. This kind of malware is very effective at rapidly infecting entire networks of systems.

### 4.3. Trojans

This kind of malware conceals itself inside or masquerades as genuine software [7]. It will break protection invisibly by establishing security holes that allow future malware versions quick access.

### 4.4. Spyware

It operates in the backdrop of a system and collects data such as credit card numbers, passwords, and other confidential material without the user's knowledge. Malware is a program that is installed directly or unintentionally on your computer [8]. Similarly to spyware, Trojan horse software is disguised as another application.

### 4.5. Ransomware

Ransomware, often known as malicious codes, leads to high costs. They can dismantle computers and prevent users from accessing them until a ransom has been paid; ransomware has attacked a few of the world's largest organizations.

### 4.6. Adware

Adware is harmful software that causes your website to navigate to online ads, many of which attempt to load additional, dangerous software.

### 4.7. Rootkit

A root kit is a program or, more often, a set of programming applications that help a threat actor remotely control and utilize a machine or even other system [9]. Its name comes from the fact that this is a suite of techniques that let attackers to obtain root privileges to a target machine and then utilize that privilege to conceal their activity.

### 4.8. Cryptojacking

Cryptojacking is the practice of mining cryptocurrency on another company server without their consent. Crypto seems to be another way for hackers to force you into giving them with Bit coin, but this occasion does so without your awareness [12]. Cryptocurrency trading software accesses your computers and uses someone computer's capabilities to mine Bit money for the attacker's profit. The mining software may run in the background of your OS or as jQuery in a web page.

### 4.9. Malvertising

Malvertising is the technique of exploiting legitimate ads or sponsored links to deliver malware covertly to the computers of unsuspecting consumers. For example, a malicious user may pay for something like the placement of an advertising on a website address [16]. When a user clicks on an advertising, the advertisement's computer either directs the user to a malicious link or infects their attacker successfully. In certain cases, spyware included with the advertising may execute automatically alone without user's involvement, a practice that is known as cars driving deployment.

### 4.10. Stealth Malware

A stealth virus is a kind of computer infection that operates in the background and avoids detection by standard antivirus or antimalware software. Stealth viruses conceal themselves in documents, sectors, and boot sectors and are skilled at secret communications. Stealth virus comes in a variety of flavors, depending on what they conceal [8]. Stealth spyware makes advantage of the attaching method to redirect the previous system call to it.

## 5. Category of Malware

**Figure 2:**
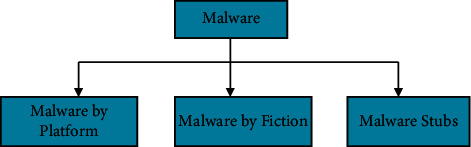
Categories of malware.

Malware is an abbreviation for malicious software that is purposefully intended to disrupt the device's operation and cause damage to the system, portable devices, websites, network, and other machines. Regrettably, malware authoring is a lucrative industry that contributes to the issue of virus proliferation [13]. As a result, hundreds of new malware types are released daily. Malware is classified into three categories, as shown in Figure 2.

### 5.1. Malware by Platform

**Figure 3:**
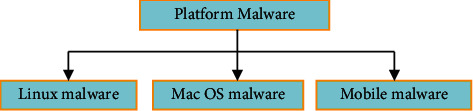
Platform-based malware.

Each and every day, a new kind of malware is launched for a different platform. Malware is further divided into four types, as shown in Figure 3, depending on the platform.

#### 5.1.1. Linux Malware

Originally, it was assumed that Linux, like the UNIX system, was less susceptible to malware [14]. However, in 2008, it was observed that the amount of malware attacking Linux was quickly rising. According to Sean Coarsen, a leading data analyst at Kaspersky, “the rise of Linux malicious programs is simply a consequence of the OS system's increasing usage, particularly as a desktop environment.” The popularity of a hardware platform is inextricably conducted in an attempt of virus writers to produce malicious files for it. Linux malware is classified into six categories, which are shown in Table 1 along with their associated purposes.

#### 5.1.2. Mac OS Malware

Historically, malware or virus attacks on Mac OS X and OS X were uncommon; therefore, MacOS was regarded less susceptible than Windows [17]. The situation has changed; MacOS virus now affects Apple's current Macintosh operating system, macOS. Malware for MacOS comprises Trojan horses, worms, and viruses, which are listed in Table 2 along with their purposes.

#### 5.1.3. Mobile Malware

Mobile malware is malicious software that directly targets electronic tools (PDAs) and mobile phones. This virus causes the system crashes due to the loss or leaking of sensitive data. Mobile malware includes a variety of malware attacks, as shown in Table 3.

### 5.2. Malware Stubs

Malware stubs cover about 170 malware types, each of which is categorized according to its behavior. Many of them seem to be simply variants of earlier malware, produced by attackers by simply altering a tiny piece of previous malware's code, referred to as a mutations; a list of malware stubs is provided in Table 4.

### 5.3. Malware in Fiction

Malware is a critical component of the work of fiction. The fiction may take the form of music, video, game, or another medium; malware, like as viruses, also plays a major part. In fiction, attackers uncover a variety of viruses, some of which are mentioned in Table 5.

## 6. Malware Detection Using Artificial Intelligence Approaches

**Figure 4:**
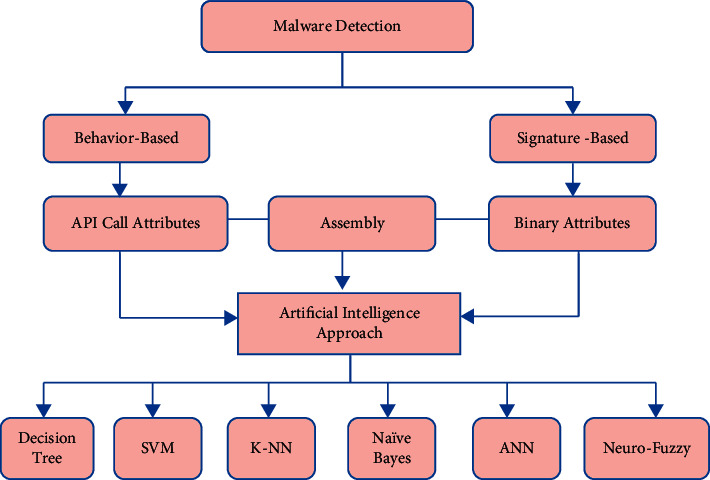
Malware detection methods.

Various artificial intelligence methods were used to recognize and classify malware, as shown in Figure 4. The graph shows how binary characteristics, assembly features, and API call attributes are all lasting malware detection techniques [18]. These factors make use of artificial intelligence techniques to deduce and detect harmful activity.

### 6.1. Signatures-Based Malicious Software Detection

Actually, static analysis signatures became mostly frequently used technique in antiviral software with concrete linkages. Instead of focusing on element behavioral methods, malware detection has mainly relied on static exams to assess the software outcome of infestations. The signature-based system identifies disruptions by referring to a previously defined database of known assaults. Although this technology can detect malware in a variety of configurations, it requires frequent updating of the previously built signature database. Additionally, the signature-based approach is less effective for identifying malicious activity due to the constantly changing intensity of transportable malware. Signature-based systems rely on extraordinary unpolished bit patterns or conventional explanation, referred to as impressions that were created to arrange the malicious document [19]. In particular, to detect whether they are defined in the project management malicious files, the static properties of the material are examined. Signature-based methods track all potential available options for an addressable document, which is the primary benefit referred to as completeness. Within the malware structure, persistent harmful products include that this would be used to create new sophisticated signatures [20]. Progovernment software vendors use meta-heuristic methods to constructively analyze the virus and limit its signature. The enormous variety of signatures is included into relevant databases used to categories malware. Rapidly identifiable proofs, ease of use, and complete openness are only a lot of small features of signature-based malware identification. Additionally, since the digitally signed methods were developed in response to prior criminal activity, particular techniques are often recognized [21]. As a result, software developers may effectively circumvent them via the use of direct distraction methodologies.

**Figure 5:**
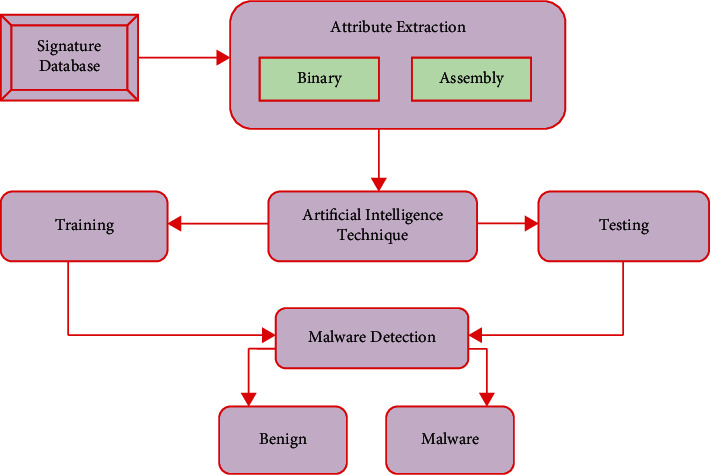
Structure of signature-based malware detection.

Furthermore, malicious code may be altered to circumvent signature-based recognition. Due to the fact that antiviral vendors built their products based on earlier identified harmful code, they are still unable to identify new malicious code or variants of existing malware. Similarly, without a genuine digital signature, signature-based methodologies can not identify polymorphism malware adequately [22]. In this manner, a signature-based identifying scheme does not provide zero-day protection. Because each malware version has a unique identifier, the signature database increases rapidly. Binary features and assembly features are the two primary methodologies used by machine learning techniques for virus detection. The structure shown in Figure 5 is a typical signature-based malware identification framework that incorporates artificial intelligence techniques.

### 6.2. Behavior-Based Malware Detection

**Figure 6:**
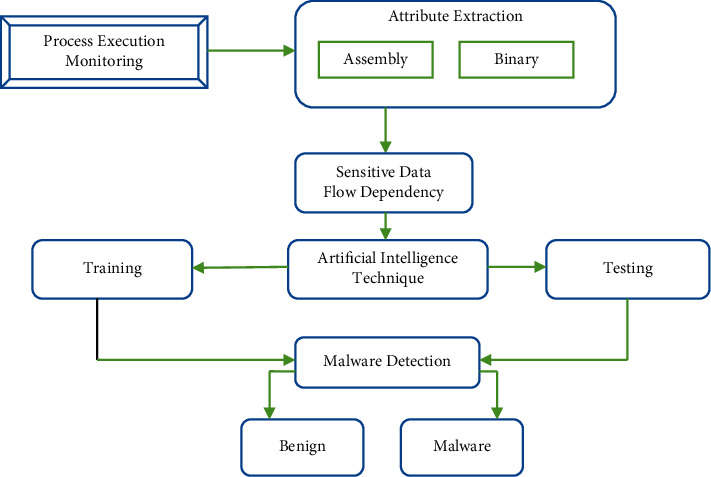
Behavior-based malware detection.

Dynamic analysis traces the virus's activity by executing the malware executable for many minutes in a virtual sandboxed environment. The most commonly utilized technique is monitoring at the system-call level [23]. A sequential report of the malware binary's observed activity is based on the malware's operations and activities. Typically, the report contains all system calls and their arguments, which are recorded in a format optimized for behavior-based analysis. Another method, dubbed forensic snapshot comparison, is based on a comparison of pre- and postinfection systems photographs. A significant distinction between system-call monitoring and forensic comparison is that the latter does not account for the glass transition of forensics occurrences. While dynamic analysis is advantageous, it is inefficient due to the malware's need to watch for many minutes. Additionally, some modern malware is intended to be “Virtual Machine” and will refrain from doing harmful actions if a virtual machine is identified [24]. The execution of an assigned model in a sandboxed environment is required for behavior-based methods, and run-time practices are observed and recorded. Dynamic assessment framework uses both virtualization and emulation to eliminate their exercises. Malicious behavior is defined as doing a thorough study of the product's code and architecture to determine spiteful expectations. API calls and group highlights are two primary methods for implementing AI calculations in behavior-based recognition.  Figure 6 illustrates a typical conduct-based malware location technique that makes use of information.

## 7. Attribute Extraction Methods

The image retrieval process was conducted after the identification of n-gram information in the very first phase. At this step, most relevant features were chosen, and the best one was evaluated using classification performance calculations that were proportionate to the set of information selected using different feature extraction methods [25]. CFsSubset, Principal Components, GainRatioAttribute, and SymmetricalUncertAttribute were utilized to choose features in this study.

### 7.1. Correlation-Based Feature Selection (CFsSubset)

The disadvantage of univariate filters, such as information gain, is that they do not account for element interaction, which is addressed by multivariate filtration, such as CFS. CFS determines the value of a selection of characteristics by taking into account each format's unique predictive ability as well as the degree to which they are redundant. Connection coefficients are now used to determine the degree of association between selections of qualities and classes, including between aspects. The significance of a variety of attributes rises with increasing correlations among variables and subclasses and decreases with increasing intercorrelation [9]. To identify the optimum segmentation results, CFS is used in combination with many other search techniques such as front selection, stepwise regression, macro and micro search, greatest browse, and genomic look online. (1) contains the CFS equations.

(1) $$\begin{array}{c} r_{zc}=\frac{k\bar{r_{zi}}}{\sqrt{k+k\left(k-1\right)\bar{r_{ii}}}}. \end{array}$$

Here, *r*_*zc*_ is the relationship seen between summing feature selection methods and the class parameters, *k* is the amount of individual difference variables, rzi is the average of the connections between individual difference variables and the category variables, and rii is the average of the system among individual difference variables [4].

### 7.2. Principal Components Analysis (PCA)

PAC is a popular used method for sparse representation in machine learning and encoding. It is based on the conversion of a significant proportion of feature extraction just the way of discovering a few complimentary nonlinear functions of it like the predictive datasets with the largest effect [26]. The first constituent of the modernization is the combination of two separate dependent variable with the greatest variance; the coefficient of multiple determinations is the set of predefined variables with both the second greatest variance that is orthogonal to the first parameter; and so forth. In many data sources, the first few main constituents account for the majority of the variation in the entire dataset, allowing the remainder to be ignored with little volatility loss during information feature reduction. PCA has been used effectively in a variety of fields, including facial recognition software, computer vision, text classification, and gene expression research. The change proceeds in the following manner. Assume that a collection of measurements *x*_1_, *x*_2_,…, *x*_*n*_ is expressed by a row vectors of length m. Thus, a matrix *X*_*n-m*_ is used to describe the set of data.

(2) $$\begin{array}{c} X_{n\times m}=\left[\begin{array}{c} x_{11}x_{12}\ldots x_{1m}\\ x_{21}x_{22}\ldots x_{2m}\\ x_{n1}x_{n1}\ldots x_{nm} \end{array}\right]=\left[X_{1},X_{2},\ldots X_{n}\right]. \end{array}$$

The term “average observations” refers to the process of determining what constitutes a reflection:

(3) $$\begin{array}{c} \mu =\frac{1}{n}\sum_{i=1}^{n}x_{i}. \end{array}$$

The deviation of a measurement from the mean is described as

(4) $$\begin{array}{c} \Phi_{i}=X_{i}-\mu . \end{array}$$

The dataset's observed covariance matrices are denoted by

(5) $$\begin{array}{c} c=\frac{1}{n}\sum_{i=1}^{n}\left(X_{i}-\mu \right){\left(X_{i}-\mu \right)}^{T}=\frac{1}{n}{\text{sum}}_{i=1}^{n}\Phi_{i}\Phi_{i}^{T}=\frac{1}{n}AA^{T}, \end{array}$$

where *A*=[Φ_1_, Φ_2_,……Φ_*n*_].

To do PCA, the Singular Value Decomposition (SVD) theorem is often used to calculate the values and associated eigenvectors of the cumulative distribution function *C*. Assume that (*k*_1_, *u*_1_), (*k*_2_, *u*_2_),, and (*k*_*m*_,*u*_*m*_) are m pairs of eigenvalues and eigenvectors for the sample covariance matrix *C*. We just choose k eigenvectors with the greatest Eigen values [27]. Usually, there will only be a few significant eigenvalues, implying that *k* is the intrinsic size of the subspace controlling the “signal,” whereas the other (*m*-*k*) dimensions are often filled with noise. The dimensions of the subdomain k may be calculated using the following formula:

(6) $$\begin{array}{c} \frac{\sum_{i=1}^{k}\lambda_{i}}{\sum_{i=1}^{m}\lambda_{i}}\geq \alpha , \end{array}$$

where a denotes the ratio of subdomain variability to the observed variability in the original space. If it is set to 99.9 percent, the variety in the region covered by the earlier *k* eigenvalues result in a loss equal variation of just 0.1 percent in the original set. We create an *m*-*k* matrix *U* with the *k* eigenvectors as columns. The highest importance description of data entails projection all data over *k*-dimensional domain using the following criteria:

(7) $$\begin{array}{c} y_{i}=U^{T}\left(X_{i}-\mu \right)=U^{T}\Phi_{i}. \end{array}$$

### 7.3. Gain Ratio Attribute

A decision tree is a straightforward structure derived from nonnodes that indicate tests across one or more characteristics and network interface layer that provide decision results. At each node of the logistic regression, the information gain measure is utilized to choose the test attribute [28]. They obtained the following metric favors characteristics with a high number of possible values. C4.5 improved the fundamental decision tree method ID3 [5]. C4.5, the successor to ID3, makes use of a mutual information augmentation known as voltage gain to try to address this bias. WEKA [15] has their own version of C4.5, and dubbed J4.8. J4.8 was used to determine the important characteristics. Assume that S is a collection of s data samples classified into m different classes. The information that is anticipated to be required to categorize a given sample that is provided by

(8) $$\begin{array}{c} I\left(S\right)=-\sum_{i=1}^{m}p_{i}\log_{2}\left(p_{i}\right). \end{array}$$

And pi denotes the probability that a representative selection belongs to the Ci class, which is computed using si/s. Consider that *A* has *v* distinct values. Allow *s*_*ij*_ to indicate the decimal digits of Ci questions included from the inside of a subgroup *S*_*j*_. *S*_*j*_ is a subset of *S* that contains samples with the frequency *a*_*j*_ of *A* [29]. The unpredictability, or expected understanding as a consequence of *A*'s subgroup categorization, is calculated as follows:

(9) $$\begin{array}{c} E\left(A\right)=-\sum_{i=1}^{m}I\left(S\right)\frac{s_{1i}+s_{2i}+\ldots s_{mi}}{s}. \end{array}$$

The data gleaned by stepping on *A* is encoder material.

(10) $$\begin{array}{c} \text{Gain}\left(A\right)=I\left(S\right)-E\left(A\right). \end{array}$$

C4.5 makes use of a gain ratio to normalize selected features using an expert analysis as

(11) $$\begin{array}{c} {\text{splitinfo}}_{A}\left(S\right)=-\sum_{i=1}^{v}\left(\frac{\left|s_{i}\right|}{\left|s\right|}\right)\log_{2}\left(\frac{\left|s_{i}\right|}{\left|s\right|}\right). \end{array}$$

The preceding number reflects the evidence received by partitioning the trained model *S* into *v* segments according to v results of a property *A* test.

The ratio of gain to loss is expressed as

(12) $$\begin{array}{c} \text{Gain Ratio}\left(A\right)=\frac{\text{Gain}\left(A\right)}{\text{SplitInfo}A\left(S\right)}. \end{array}$$

The dividing characteristic with the greatest gain ratio is chosen [1]. The nonleaf nodes of the produced decision tree are regarded as significant characteristics [30]. The authors combined decision trees and neural networks, resulting in an increase in classification results.

### 7.4. SymmetricalUncertAttributeEval

SymmetricalUncertAttributeEval evaluates characteristics in terms of their symmetry ambiguity. The number of the SymmetricalUncertainAttributeEval properties is either 0 or only one, with one indicating that the characteristic or property is important to the class and zero indicating that it is unimportant.

## 8. Methodology

Identification is a learning process in which the classifier gains knowledge from labeled samples. The algorithm is then evaluated for recognition rate using raw data. Each instance in the test dataset is associated with a single target value and a number of characteristics. The whole procedure includes the categorization of unidentified files as dangerous or benign using machine learning techniques. There are 2 different methods: training phase. During the learning phase, the software is fed a learning algorithm of legitimate and malicious files. The algorithm for learning develops a classifier. Even during testing process, the classifier classifies a selection of harmful. The ANN, SVM, J48, and NB classifiers were employed in this study. Neural networks operate in a manner similar to that of the nervous system. The concept of a human brain is discussed in detail in [9, 25].

### 8.1. Artificial Neural Network

**Figure 7:**
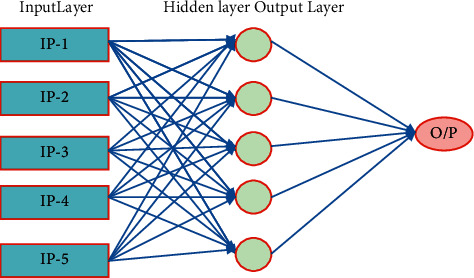
A typical architecture of ANN.

An ANN [22] is a paradigm for data processing influenced by the ways particular human minds, including the brains, interpret things. The critical aspect is the knowledge acquisition system's organization, which is a network consisting of millions of densely linked neurons cooperating to mimic a certain function, as illustrated in Figure 7. An ANN is customized for a particular function, such as analytical thinking or data categorization, through an education process where the constituent strengths of the various neurons inputs are changed using a training method such as home. Weights are changed in real time in response to the examples received by the networks, thus reducing the output error. The output calculation of a multiple ANN is shown in formula (13), where *x* is just the input image, *v*_*i*_ is the frequency of the output unit, *g* is the algorithm, *w*_*ij*_ is the weighting of such a hidden neuron, and *b*_*i*_, o is a bias.

(13) $$\begin{array}{c} f\left(x\right)=g\left[\sum_{i}v_{i}g\left[\sum_{j}W_{ij}x_{j}+b_{j}\right]+\;b_{0}\right]. \end{array}$$

### 8.2. Decision Trees

The decision tree operator [24] is a well-known class of machine learning. Approaches are shown as trees with individual nodes representing labeled data tests and leaves representing categorization judgments (classes). Traditionally, a greedy met heuristic optimization technique is employed to create a tiny decision tree that is produced from the training dataset through variable partitioning based on anticipated classification algorithm. This technique classifies the experimental data properly. Pruning is a feature of modern systems that eliminates the issue of overfitting [31]. We utilized J48, the Weka [24] implementation of the C4.5 method [23]. A distinguishing feature of deliberation trees is their clear system of representation, which may be readily expressed as rules.

### 8.3. Support Vector Machine (SVM)

**Figure 8:**
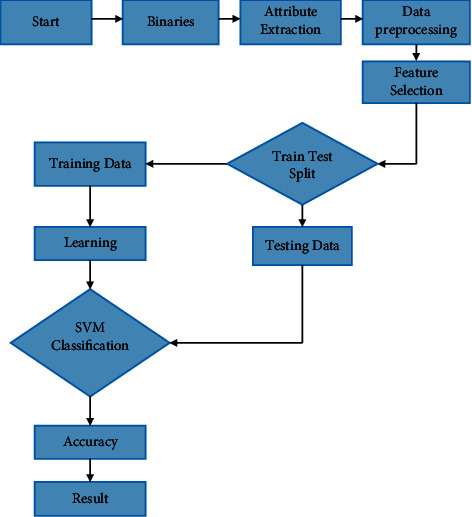
Flow diagram for malware detection and classification using artificial intelligence techniques.

A SVM is a one-of-a-kind classifier that is described mathematically by a higher dimensional space. Essentially, in Figure 8. SVM, distinct lines, or graphs are utilized to classify or discriminating between distinct classes or instances. Class division is what SVM does. As with kernel regression analysis, it draws lines to denote classifications.

SVMs are a kind of kernel classification algorithm. Furthermore, the SVM employs a goal that expressly promotes excellent classification performance. Because SVMs do not fit well inside a Bayesian manner, we will discuss them quickly here [6]. Given that Kolter and Maloof employed SVM and had favorable results, referencing those locations may provide favorable results for us as well. In the SVM research, the terms +1 and −1 are often used to represent the 2 classes. A linear differentiator is defined as wTx + *b* ≥ 0 class +1, 0 class −1 for a dimensional space characterized by respect to the weights and bias b. traditionally, we utilize SVM because when the model is trained, it is not too large.

### 8.4. Naïve Bayes

The Naive Bayes scheme was based upon that Naive Bayes algorithm, which says that the likelihood function of a class is proportionate to its highest distribution and to the conditioned possibility of the characteristics given this class inside this context of categorization. A Bayes procedure must calculate posterior probability for an exponentially number of components configurations if no independence questions are raised [11]. Naive Bayes optimizes this procedure by considering that properties are random variables with respect to the class and requiring the estimation of just a linear set of variables. The prior probabilities for every class and indeed the possibility of each aspect supplying each class may be readily calculated from classification model and used to calculate the posterior accuracy of the classifier given a collection of features. Naive Bayes has already been shown scientifically to correctly categorizes data across a wide plethora of different areas [25].

## 9. Evaluation Criteria

The efficiency of each classification was determined using all attribute methodologies and the appropriate amount of features chosen through image segmentation. The studies were assessed using TPR, TNR, FPR, and FNR, as described by [9].

### 9.1. True Negative Rate

TNR is the proportion of benign sample properly classified as negatives or false.

(14) $$\begin{array}{c} \text{TNR}=\frac{\left(\text{TN}\right)}{\left(\text{TN}+\text{FP}\right)}. \end{array}$$

### 9.2. False Positive Rate

The FPR is the proportion of nonmalware samples that are erroneously detected as malicious.

(15) $$\begin{array}{c} \text{FPR}=\frac{\left(\text{FP}\right)}{\left(\text{FP}+\text{FN}\right)}. \end{array}$$

### 9.3. True Positive Rate

The TPR is the proportion of genuine positives that are properly classified as malicious activity.

(16) $$\begin{array}{c} \text{TPR}=\frac{\left(\text{TP}\right)}{\left(\text{TP}+\text{FN}\right)}\text{.} \end{array}$$

### 9.4. False Negative Rate

The FNR is the proportion of malware that have been erroneously classified as harmless.

(17) $$\begin{array}{c} \text{FNR}=\frac{\left(\text{FN}\right)}{\left(\text{FN}+\text{TP}\right)}\text{.} \end{array}$$

### 9.5. Accuracy

The proportion of correctly classified objects is used to calculate overall effectiveness (true positives and negatives).

(18) $$\begin{array}{c} \text{Accuracy}=\frac{\left(\text{TP}+\text{TN}\right)}{\left(\text{TP}+\text{TN}+\text{FP}+\text{FN}\right)}\text{.} \end{array}$$

Here, TP, TN, FP, and FN are defined as follows:

- TP: total number of malicious files that have been verified accurately as malicious files
- TN: the total number of mild files that have been clearly predicted as benign files
- FT: the number of malicious files that have been misidentified as nonmalware files
- FN: the number of nonmalware files that have been mislabeled as malicious files

## 10. Results and Discussion

Tables 5 and 6 describe the training and validation processes. The findings of the research to use the whole data to train are summarized in Table 5, while the findings of the research using 80% of the original dataset with learning and 20% of the information for assessment are summarized in Table 6. The NN, SVM, and J48 algorithms performed the best in terms of effectiveness and false positive. NB had a poor accuracy rate and a significant rate of false positives. This suboptimal effectiveness was caused by the Naive Bayes' assumptions of component interdependence. Among all background subtraction types, the classification algorithm achieved the best results. When the precision of the classification was measured by the amount of features chosen by the segmentation techniques, clearly, the SVM and PCA classifiers performed best only with minimum characteristics. Notably, the Neural Network classification failed to function as the number of attributes increased, as shown in Table 7 by the Association Characteristic feature extractor. Although CFsSubset produced the fewest features, its accuracy was inferior to that of the other feature extraction techniques. The Info Gaining Attributes Evaluator, Correlation Argument Evaluator, Gain Ratio Attribute Evaluator, and Rotationally symmetric Uncert Attributes Evaluator all achieved highly accurate but chose a greater variety of components than the PCA, which evaluated a limited list of advantages with great accuracy. As a result, the PCA demonstrated to be the most effective method for selecting significant characteristics for categorization.

**Figure 9:**
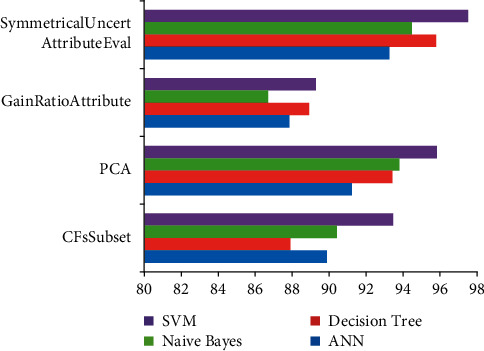
Accuracy ratios for classification techniques with various segmentation techniques using an entire training sample.

The performance ratio for classification techniques with various segmentation techniques utilizing the intensive training dataset is shown in Figure 9. The NN, SVM, and J48 algorithms performed the best in terms of effectiveness and false positive. NB had a low degree of precision and a high percentage of false positive. This suboptimal effectiveness was caused by the Naive Bayes' presumption of feature independent. The SVM had the best accuracy across all subset of features methods.

**Figure 10:**
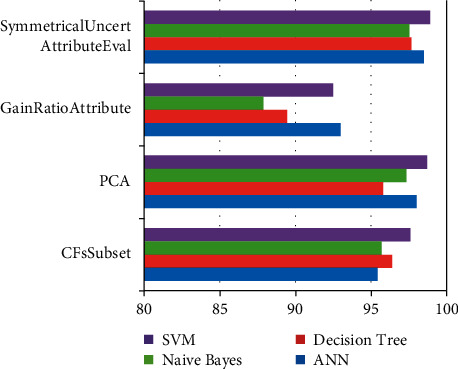
Different techniques for selecting features in relation to classification techniques that use 80% of the information for trained and 20% for tests.

The efficiency ratios for classification techniques with various feature extraction techniques were calculated using 80% of the information for trained and 20% for testing as shown in Figure 10. NN, SVM, and J48 showed the greatest effectiveness with a low error rate. NB had a poor accuracy rate and a significant rate of false positives. This suboptimal effectiveness was caused by the Naive Bayes' assumptions of features dependence. The classifier had the best accuracy across all feature extraction types.

## 11. Conclusion

This research investigated the detection and tracking of malware, as well as attribute retrieval utilizing machine learning and artificial intelligence. The studies showed that when PCA was used for feature extraction, it resulted in a significant decrease in the number of characteristics for malicious apps when comparing to other techniques. Additionally, the PCA needed minimal training and outperformed the other methodologies. A high degree of accuracy was created by incorporating PCA and SVM with the whole dataset being utilized for training. Additionally, the results indicated that the SVM was remarkably precise, which was consistent with the findings of other studies. This demonstrates that this kind of classifier can produce the best results with a high degree of accuracy. In the approach, the same methods may be used to identify more sophisticated malicious files (compostable and transformational). Additionally, it will be fascinating to evaluate the efficacy of other deep learning approaches for attack detection. In the future, new methods and effort will be required. This article will assist in comprehending the methodological approaches for malware to present and therefore serve as a useful reference for future studies.

## Figures and Tables

**Table 1:** List of MacOS malware.

S. no.	Name of the malware	Function
1	Devnull	It downloads and executes commands, followed by compressed executable code.
2	Hajime	It makes an effort to secure assets.
3	Linux Spike Trojan	It infiltrates networks and ultimately spreads to certain other networks, resulting in DDoS assaults, MITM attacks, data theft, as well as other kinds of attacks.
4	Linux.Daelloz	By abusing PHP, It infects networks, video surveillance, and set-top boxes.
5	Staog	It uses kernel flaws to get access to residents.
6	Mirai	It is capable of infecting consumer electronics including Webcams and edge routers.

**Table 2:** List of MacOS malware.

S. no.	Name of the malware	Function
1	Fake flash	It attacks Apple Mac systems via Flash Player and other False Adobe Trojans.
2	Genieo	Genieo injects adware and user monitoring software.
3	KeRanger	It is implemented through with vulnerability in Communication that was concealed inside the.dmg file under General.rtf. More than 9500 mac users were impacted.
4	Mac Defender	It is a rogue security problem in the form of bogus antivirus.
5	MacSweeper	It confuses consumers by exaggerating reports of malware, adware, or viruses.
6	OSX.Keydnap	It steals credentials from the compromised device's iCloud Account.

**Table 3:** List of mobile malware.

S. no.	Name of the malware	Function
1	Brain Test	It represents a new degree of sophistication in malware.
2	Dendroid	It attacks the mobile phone and damages the Android operating system.
3	Shedun	It affects over 3000 Android apps.
4	KeyRaider	The iPhone is targeted, the machine is locked, the user's username and password are stolen, and the user is demanded to pay a ransom. Malware affects almost 3,36,000 people.

**Table 4:** List of malware stubs.

S. no.	Name of the malware
1	Acid
2	Acme
3	Backoff
4	Bohmini.A
5	Coreflood
6	Dexter
7	FastPOS
8	Doppelganger domain

**Table 5:** List of malware in fiction.

S. no.	Name of the malware	Function
1	Digimon Adventure	The documentary follows a group of youngsters who are transferred to another digital world and charged with defending it from evil.
2	Digimon Adventure	
3	Digimon Adventure tri	
4	Digimon D-Cyber	
5	Digimon Data Squad	
6	Digimon Fusion	

**Table 6:** Various attitudes for selecting features in relation to classification techniques for 100% training.

Attribute extraction	No. of attributes	ANN (Acc, TPR, FPR, TNR, FNR) (%)	Decision tree (Acc, TPR, FPR, TNR, FNR) (%)	Naive Bayes (Acc, TPR, FPR, TNR, FNR) (%)	SVM (Acc, TPR,FPR, TNR, FNR) (%)
CFsSubset	25	89.9	91.3	87.9	93.3
		100	100	100	100
		11.9	13	15	10
		86.8	88	88.9	90
		0	0	0	0

PCA	55	87.9	93.5	88.9	94.8
		63.8	77	74	96
		5	4	11	6
		94.8	97	91	98
		34.7	22	25	0

GainRatioAttribute	201	90.5	93.8	86.7	94.5
		1	100	100	100
		9	10	17	8
		90	89	86	91
		0	0	0	0

SymmetricalUncertAttributeEval	146	93.5	93.8	89.3	95.5
		100	100	100	100
		9.9	10	11	8
		88.7	89.6	83	91
		0	0	0	0

**Table 7:** Different techniques for selecting features in relation to classification techniques that use 80% of the information for trained and 20% for tests.

Attribute extraction	No. of attributes	ANN (Acc, TPR, FPR, TNR, FNR)	Decision tree (Acc, TPR,FPR, TNR, FNR)	Naive Bayes (Acc, TPR, FPR, TNR, FNR) (%)	SVM (Acc, TPR, FPR, TNR, FNR) (%)
		(%)	(%)		
CFsSubset	25	95.4	97	93	98.5
		100	100	100	100
		3	6	9	3
		94	96	89	96
		0	0	0	0

PCA	55	96.4	95.8	91.5	97.7
		100	93	93	91
		4	7	9	0
		95	98	88	98
		0	7	6	8

GainRatioAttribute	201	95.7	97.3	87.9	97.5
		100	100	100	100
		6	3	13	5
		8	97	84	97
		0	0	0	0

SymmetricalUncertAttributeEval	146	94.6	94.3	90.5	97.9
		100	100	100	100
		5	6	12	3
		94	93	85	96
		0	0	0	0

## Data Availability

The datasets used and/or analyzed during the current study are available from the corresponding author upon reasonable request.
